# An investigation of the effects of lipid-lowering medications: genome-wide linkage analysis of lipids in the HyperGEN study

**DOI:** 10.1186/1471-2156-8-60

**Published:** 2007-09-10

**Authors:** Jun Wu, Michael A Province, Hilary Coon, Steven C Hunt, John H Eckfeldt, Donna K Arnett, Gerardo Heiss, Cora E Lewis, R Curtis Ellison, Dabeeru C Rao, Treva Rice, Aldi T Kraja

**Affiliations:** 1 Division of Statistical Genomics, Washington University School of Medicine, Campus Box 8506, 4444 Forest Park Boulevard, Saint Louis, MO63108, USA; 2 Department of Psychiatry, University of Utah, Salt Lake City, UT, USA; 3 Cardiovascular Genetics, University of Utah, Salt Lake City, UT, USA; 4 Department of Laboratory Medicine & Pathology, University of Minnesota, Minneapolis, MN, USA; 5 University of Alabama at Birmingham, Birmingham, AL, USA; 6 Department of Epidemiology, University of North Carolina, Chapel Hill, NC, USA; 7 Division of Preventive Medicine, University of Alabama at Birmingham, Birmingham, AL, USA; 8 Section of Preventive Medicine and Epidemiology, Boston University School of Medicine, Boston, MA, USA; 9 Division of Biostatistics, Washington University School of Medicine, Saint Louis, MO, USA

## Abstract

**Background:**

Use of anti-hyperlipidemic medications compromises genetic analysis because of altered lipid profiles. We propose an empirical method to adjust lipid levels for medication effects so that the adjusted lipid values substitute the unmedicated lipid values in the genetic analysis.

**Results:**

Published clinical trials were reviewed for HMG-CoA reductase inhibitors and fibric acid derivatives as mono-drug therapy. HMG-CoA reductase inhibitors showed similar effects in African Americans (AA) and non-African Americans (non-AA) for lowering total cholesterol (TC, -50.7 mg/dl), LDL cholesterol (LDL-C, -48.1 mg/dl), and triglycerides (TG, -19.7 mg/dl). Their effect on increasing HDL cholesterol (HDL-C) in AA (+0.4 mg/dl) was lower than in Non-AA (+2.3 mg/dl). The effects of fibric acid derivatives were estimated as -46.1 mg/dl for TC, -40.1 mg/dl for LDL-C, and +5.9 mg/dl for HDL-C in non-AA. The corresponding effects in AA were less extreme (-20.1 mg/dl, -11.4 mg/dl, and +3.1 mg/dl). Similar effect for TG (59.0 mg/dl) was shown in AA and non-AA. The above estimated effects were applied to a multipoint variance components linkage analysis on the lipid levels in 2,403 Whites and 2,214 AA in the HyperGEN study. The familial effects did vary depending on whether the lipids were adjusted for medication use. For example, the heritabilities increased after medication adjustment for TC and LDL-C, but did not change significantly for HDL-C and TG.

**Conclusion:**

Ethnicity-specific medication adjustments using our empirical method can be employed in epidemiological and genetic analysis of lipids.

## Background

There is extensive epidemiological evidence showing that a dyslipidemic profile, characterized by elevated plasma levels of total cholesterol (TC), triglycerides (TG) and LDL cholesterol (LDL-C), and reduced levels of HDL cholesterol (HDL-C), is strongly associated with an increased risk of atherosclerosis and coronary heart disease [1]. Genetic contributions to plasma lipid levels also have been documented, with the magnitude as assessed by heritability being quite variable (26% to 83%) [2-4]. Similarly, genome scans from different populations have reported a variety of quantitative trait loci (QTLs) related to lipid profiles [5-13].

However, it is known that use of anti-hyperlipidemic medications by participants in such family studies distorts their blood lipid levels. Consequently, investigators may exclude subjects on medications during recruitment, or remove them at the time of the genetic analyses. Not only do such exclusions results in a smaller sample size, they also may discard the participants who are most likely to carry causative variants of dyslipidemia. To increase the power of the analysis by including these potentially informative subjects, some studies were designed to interrupt lipid lowering medication for specific time intervals before the blood samples were taken. Other methods that have been implemented are to adjust the lipid levels for medication by using medications as covariates in a linear regression model. This method tends to minimize the differences in lipid levels between medicated and unmedicated groups, but not necessarily adjust lipid levels toward their original values. To our knowledge, only one study has performed a genome-wide scan for LDL-C after increasing the measured levels by 25% for medicated subjects [13]. The lod score for their highest peak increased from 3.27, observed when analyzing 1977 white subjects not on lipid lowering medications, to 3.72 after including 350 medicated subjects.

The aim of the present study is to recover or impute the original unmedicated lipid levels using an empirical approach. The measured (biased) lipid values of individuals on lipid-lowering drugs are "adjusted" by increasing (or decreasing) the measured value by the same amount that clinical trials studies report decreases (or increases) for a given class of drugs using ethnic-specific data. That is, the approach statistically infers or projects what the original lipid value would have been prior to medication treatment. The impact of the medication adjustments will be evaluated by computing heritabilities and by conducting genome-wide linkage analyses in three samples: by excluding medicated subjects; by including medicated subjects after adjusting lipid values for medication use; and by including medicated subjects without any medication adjustment. We hypothesize that imputing the original lipid measurement by adjusting for medication effects can provide more precise estimates of the medication-free values of the phenotypes, with an expected increase in power to detect genes involved in the lipid metabolism.

## Methods

### Subject description

The Hypertension Genetic Epidemiology Network (HyperGEN) study is part of the National Heart, Lung and Blood Institute (NHLBI) Family Blood Pressure Program. The design and methods have been described in detail elsewhere [14]. In the present report 2,403 White and 2,214 African American participants represent 1,924 pairs of White hypertensive siblings in 1,242 nuclear families and 1,889 pairs of African American hypertensive siblings in 1,801 nuclear families. Among all participants with measured serum lipid levels, 402 subjects reported that they were taking medications to lower blood lipid levels. This included 385 subjects taking one medication, 16 subjects on combination drug therapy of two medications, and 1 subject reporting the use of three drugs. IRB approvals were obtained for all participating centers, and all participants gave their written informed consent.

### Measurement of Lipids and Other Phenotypes

All blood assays on samples were performed at the Central Biochemistry Laboratory at the University of Minnesota. TG levels were colorimetrically determined by using a peroxidase-coupled method on a centrifugal analyzer [15]. TC was measured by using a commercial cholesterol oxidase method [16]. LDL-C was estimated by the Friedewald equation [17], or by ultracentrifugation for subjects with TG above 400 mg/dl [18]. HDL-C was quantified by COBAS FARA (Roche Diagnostic Systems) after precipitation of the other lipoprotein fractions by dextran sulfate [19].

Anthropometric measurements were collected with subjects wearing lightweight suits. Body mass index (BMI) was subsequently calculated as weight in kilograms divided by height in meters squared. Waist circumference was measured to the nearest centimeter at the level of the umbilicus, and hip circumference was measured at the level of the maximal circumference of the gluteus. All other measurements were collected using a common, standardized protocol administered by trained personnel.

### Genotyping

The NHLBI Mammalian Genotyping Service (Marshfield, Wisconsin) genotyped 391 markers at approximate 10-cM intervals throughout the genome, with an average marker heterozygosity of 76%. Detailed information on the genotyping methods, instruments, and software used can be found at the Marshfield Laboratory World Wide Web site [20]. Quality control of the markers and the relationships in the pedigrees were carried out at the HyperGEN Data Coordinating Center at Washington University in Saint Louis, by using ASPEX [21], GRR [22], MAPMAKER/SIBS [23], and PEDCHECK [24].

### Adjustment for the effects of lipid-lowering medications

The majority of the participants in the HyperGEN study, that were using anti-hyperlipidemic medications, were prescribed two groups of medications: HMG-CoA reductase inhibitors (statins) and/or fibric acid derivatives (Table 1). Therefore, we only focused on lipid-lowering effects of these two groups of medications. Clinical trials on the long-term effects of mono-drug therapy using these anti-hyperlipidemic medications published in English during 1989–2003 were searched online through PubMed. The search terms used included lipids, efficacy, mono-drug therapy, combination drug therapy, as well as the names of medication classes. We excluded clinical trials with duration of less than 8 weeks and those without placebo control. Clinical trials for medication combinations were also excluded unless they reported the data for mono-drug therapy as well. Tables A1 and A2 in Appendix A [see Additional File 1] summarize 32 clinical trials that have investigated the effects of HMG-CoA inhibitors and fibric acid derivatives as mono-drug therapy with commonly used dosages. A total of 19,384 participants, aged 18 to 80 years, with high blood lipid levels were pooled. The clinical trials were double-blinded, randomized with a course which varied from 8 to 280 weeks. Gemfibrozil was the only fibric acid derivative used by the subjects in the HyperGEN study; this medication was well represented in non-African Americans of the HyperGEN, but only two African Americans used the drug (Table 1). Therefore we reviewed only the clinical trials for the effects of this medication in non-African Americans (see Additional File 1 Table A2).

**Table 1:** Mechanisms of action for the major groups of antihyperlipidemic medications and their frequency of use by the subjects in HyperGEN

**Medication group**	**Mechanism**	**Frequency of use**	
		
		**African Americans**	**Caucasians**
Bile sequestrants	A partial removal of bile acids from the enterohepatic circulation by preventing their absorption	1	12
Fibric acid derivatives	Increasing fatty acid oxidation to decrease formation of VLDL (very low density lipoprotein) triglycerides	2	39
HMG CoA reductase inhibitors	Inhibiting HMG CoA reductase, the rate-limiting step in cholesterol biosynthesis	101	264
Nicotinic acid derivatives	Inhibiting lipoprotein synthesis and decrease the production of VLDL particles	1	0

The weighted (by sample size) average absolute change for each lipid variables, which took into account placebo effect, was calculated by medication groups based on ethnicity (African Americans vs. non-African Americans, and vs. all ethnicities) (Table 2). Each phenotype of lipids responded to different classes of drugs differently, and the response also varied considerably by ethnicity. Non-African Americans included mainly Whites and Hispanics. The clinical trials for all ethnicities include more trials, because some studies had samples that could not be categorized into either African Americans or non-African Americans. HMG-CoA inhibitors showed similar reductions of TC, LDL-C and TG in African Americans and non-African Americans. Thus, the following effect estimates, -50.7 mg/dl, -48.1 mg/dl, and -19.7 mg/dl, were used for all ethnicities in performing the adjustments, respectively. However, non-African Americans appeared to be more responsive than African Americans to HMG-CoA inhibitors effecting HDL-C. The ethnicity specific average effects (+2.3 mg/dl for non-African Americans and +0.4 mg/dl for African Americans) were used to obtain the adjusted HDL-C. Gemfibrozil appeared to be more effective as an anti-hyperlipidemic agent for TC, LDL-C, and HDL-C in non-African Americans than in populations with all pooled ethnicities. For non-African Americans subjects receiving Gemfibrozil, the measured levels of TC, LDL-C, and HDL-C were adjusted by the following respective estimates: -46.1 mg/dl, -40.1 mg/dl, and +5.9 mg/dl. Instead, the measured lipid levels of two African Americans who took Gemfibrozil were adjusted by the effect for all ethnicities: -20.1 mg/dl for TC, -11.4 mg/dl for LDL-C, and + 3.1 mg/dl for HDL-C. Both African Americans and non-African Americans were adjusted by -59.0 mg/dl for TG.

**Table 2:** Average effects estimated from published clinical trails by ethnicity for HMG-CoA reductase inhibitors and fibric acid derivatives

**Medication**	**Phenotype**	**African Americans**		Non-African Americans *		**All**	
				
		**No. of pts pooled**	**Average effect (mg/dl) ± SE**	**No. of pts pooled**	**Average effect (mg/dl) ± SE**	**No. of pts pooled**	**Average effect (mg/dl) ± SE**
**HMG-CoA reductase inhibitors**	**TC**	572	-51.4 ± 11.2	10637	-52.1 ± 13.2	16811	**-50.7 ± 13.9**
	**LDL-C**	572	-47.9 ± 7.2	10637	-49.9 ± 13.6	16811	**-48.1 ± 13.9**
	**HDL-C**	572	**+0.4 ± 1.7**	10637	**+2.3 ± 0.5**	16811	+2.0 ± 0.8
	**TG**	572	-18.8 ± 4.2	10637	-18.4 ± 3.7	16811	**-19.7 ± 4.5**
**Fibric acid derivatives (Gemfibrozil)**	**TC**	-	-	477	**-46.1 ± 8.6**	2573	**-20.1 ± 15.3**
	**LDL-C**	-	-	477	**-40.1 ± 7.0**	2573	**-11.4 ± 14.4**
	**HDL-C**	-	-	477	**+5.9 ± 1.8**	2573	**+3.1 ± 1.6**
	**TG**	-	-	477	-57.1 ± 14.1	2573	**-59.0 ± 10.4**

* Non-African Americans mainly include Whites, Hispanic.

TC =Total Cholesterol; HDL-C = High Density Lipoprotein Cholesterol; LDL-C = Low Density Lipoprotein Cholesterol; TG = Triglycerides.

For the participants receiving mono-drug therapy of anti-hyperlipidemics, the levels of TC, HDL-C, LDL-C, and TG were adjusted with the effect estimates, where the sign (+/-) indicates the increasing or lowering effect of a medication to a specific lipid component. When an individual took a combination therapy of two drugs, the total effect was estimated by selecting the medication with higher effect on the lipids and conservatively adding up half effect of the other medication. Unadjusted measurements were used for 12 African Americans and 2 Whites who took Bile Sequestrants or Nicotinic Acid Derivatives. The third medication effect, which was presented for only one participant, was not considered in our study.

### Statistical Methods and Linkage Analysis

TG with and without medication adjustments were transformed using natural logarithm to an approximate normal distribution. Each phenotype was then adjusted for its mean and variance to remove the effects of age as well as the effects of BMI, current smoking and drinking status, the use of estrogen replacement therapy, diabetes status, and the field center. Stepwise multiple regressions were performed for the adjustment within each ethnicity and sex group, retaining only the terms significant at the 5% level. The adjusted variables were finally standardized to a mean of 0 and a standard deviation (SD) of 1. Four to ten outliers greater than 4 SD-s from the mean and at least 1 SD from the nearest data point were excluded within each ethnic group for the lipid phenotypes.

Heritability (h^2^), defined as the percent of variance due to additive familial effects, was estimated through the maximum-likelihood methods available in the software SEGPATH under the most parsimonious model using the following equation [25]:

h2=(rparent−offspring+rsibling)(1+rspouse)1+rspouse+2rspouserparent−offspring
 MathType@MTEF@5@5@+=feaafiart1ev1aaatCvAUfKttLearuWrP9MDH5MBPbIqV92AaeXatLxBI9gBaebbnrfifHhDYfgasaacH8akY=wiFfYdH8Gipec8Eeeu0xXdbba9frFj0=OqFfea0dXdd9vqai=hGuQ8kuc9pgc9s8qqaq=dirpe0xb9q8qiLsFr0=vr0=vr0dc8meaabaqaciaacaGaaeqabaqabeGadaaakeaacqWGObaAdaahaaWcbeqaaiabikdaYaaakiabg2da9maalaaabaGaeiikaGIaemOCai3aaSbaaSqaaiabdchaWjabdggaHjabdkhaYjabdwgaLjabd6gaUjabdsha0jabgkHiTiabd+gaVjabdAgaMjabdAgaMjabdohaZjabdchaWjabdkhaYjabdMgaPjabd6gaUjabdEgaNbqabaGccqGHRaWkcqWGYbGCdaWgaaWcbaGaem4CamNaemyAaKMaemOyaiMaemiBaWMaemyAaKMaemOBa4Maem4zaCgabeaakiabcMcaPiabcIcaOiabigdaXiabgUcaRiabdkhaYnaaBaaaleaacqWGZbWCcqWGWbaCcqWGVbWBcqWG1bqDcqWGZbWCcqWGLbqzaeqaaOGaeiykaKcabaGaeGymaeJaey4kaSIaemOCai3aaSbaaSqaaiabdohaZjabdchaWjabd+gaVjabdwha1jabdohaZjabdwgaLbqabaGccqGHRaWkcqaIYaGmcqWGYbGCdaWgaaWcbaGaem4CamNaemiCaaNaem4Ba8MaemyDauNaem4CamNaemyzaugabeaakiabdkhaYnaaBaaaleaacqWGWbaCcqWGHbqycqWGYbGCcqWGLbqzcqWGUbGBcqWG0baDcqGHsislcqWGVbWBcqWGMbGzcqWGMbGzcqWGZbWCcqWGWbaCcqWGYbGCcqWGPbqAcqWGUbGBcqWGNbWzaeqaaaaaaaa@91FD@

where r_parent-offspring_, r_sibling_, and r_spouse _denote the average parent-offspring, the average sibling and the spouse correlations, respectively. Two heritability estimates were regarded as significantly different from each other if their difference was greater than the sum of their standard errors.

Genome-wide linkage analyses, both with and without adjustments for medication effects, were performed for each lipid phenotype by using variance component linkage analysis as implemented in SEGPATH [26]. Linkage analysis was carried out separately within ethnicity by using ethnicity-specific marker allele frequencies derived from the corresponding random sub-samples. Multipoint IBD estimates were used in the linkage analysis.

## Results

Table 3 presents the selected characteristics of participants in the HyperGEN study by ethnic group and gender, and the effects of adjustment for medication for each lipid parameter. The average age was approximately 53 years in Whites and 46 years in African Americans. African American females had higher levels of BMI than the other groups. Alcohol consumption, smoking status, diabetes, estrogen use, and field center also showed significant ethnic differences. When lipid levels in subjects on lipid-lowering medications were adjusted for medication effects, there were observable elevations in the mean of TC and LDL-C, but very slight changes in the mean of HDL-C and in the log transformed TG.

**Table 3:** Characteristics of subjects by ethnicity and sex in the HyperGEN study

**Variable**	**Whites**		**African Amercians**	
		
	**Males**	**Females**	**Males**	**Females**
**Age, year (n)**	52.9 ± 14.0 (1158)	53.7 ± 13.15 (1245)	45.25 ± 13.01 (814)	46.45 ± 13.32 (1400)
**BMI, kg/m^**2 **^(n)**	29.0 ± 4.7 (1156)	29.0 ± 6.84 (1242)	29.54 ± 6.32 (813)	33.38 ± 8.14 (1399)
**Waist/hip, ratio (n)**	1.0 ± 0.1 (1104)	0.9 ± 0.09 (1158)	0.93 ± 0.07 (800)	0.88 ± 0.08 (1366)
**Drinking status, % yes (n)**	42.71% (486/1138)	31.35% (380/1212)	47.64% (383/804)	21.08% (288/1366)
**Smoking status, % yes (n)**	9.50% (108/1137)	10.55% (128/1213)	37.81% (304/804)	22.62% (309/1366)
**Diabetes, % yes (n)**	10.02% (116/1158)	10.12% (126/1245)	14.00% (114/814)	18.94% (265/1399)
**Estrogen, % yes (n)**	-	42.07% (509/1210)	-	30.65% (418/1364)
**Research center, % (n)**				
**North Carolina**	7.08% (82/1158)	12.61% (157/1245)	22.11% (180/814)	27.86% (390/1400)
**Minnesota**	28.76% (333/1158)	27.23% (339/1245)	-	-
**Massachusetts**	27.20% (315/1158)	28.59% (356/1245)	-	-
**Utah**	36.87% (427/1158)	31.41% (391/1245)	-	-
**Alabama**	0.09% (1/1158)	0.16% (2/1245)	77.89% (634/814)	72.14% (1010/1400)
**LDL-C, mg/dL (n)**	118.26 ± 30.62 (1107)	117.26 ± 33.10(1217)	119.79 ± 37.63 (790)	117.95 ± 37.00 (1376)
**LDL-C (adjusted), mg/dL (n)**	125.03 ± 32.78 (1107)	122.19 ± 36.55 (1217)	121.74 ± 39.49 (790)	120.12 ± 38.52 (1376)
**HDL-C, mg/dL (n)**	43.05 ± 10.87 (1155)	55.04 ± 15.42 (1240)	49.15 ± 14.73 (805)	55.99 ± 15.17 (1382)
**HDL-C (adjusted), mg/dL (n)**	42.62 ± 10.92 (1155)	54.78 ± 15.54 (1240)	49.13 ± 14.75 (805)	55.98 ± 15.18 (1382)
**TC, mg/dL (n)**	193.22 ± 35.84 (1155)	201.23 ± 37.97 (1240)	192.23 ± 41.67 (807)	194.20 ± 40.86 (1382)
**TC (adjusted), mg/dL (n)**	200.40 ± 38.66 (1155)	206.52 ± 42.05 (1240)	194.32 ± 43.84 (807)	196.51 ± 42.64 (1382)
* TG, mg/dL (n)	4.94 ± 0.58 (1155)	4.85 ± 0.55 (1240)	4.59 ± 0.58 (807)	4.49 ± 0.51 (1382)
* TG (adjusted), mg/dL (n)	4.96 ± 0.58 (1155)	4.86 ± 0.56 (1240)	4.60 ± 0.58 (807)	4.50 ± 0.51 (1382)

BMI = Body Mass Index; TC = Total Cholesterol; HDL-C = High Density Lipoprotein Cholesterol; LDL-C = Low Density Lipoprotein Cholesterol; TG = Triglycerides.

The effects of medication adjustments on the heritabilities of lipids are summarized in Table 4. Three groups of data were analyzed: 1) Excluding medicated participants; 2) All participants without any adjustment for medication effects; 3) All participants with adjustment for medication effects. Compared to the estimates without medication adjustment, the heritabilities in Whites improved significantly by 20% and 25% for TC and LDL-C, respectively, when medication adjustments were applied. In African Americans this significant improvement was 36% for TC and 27% for LDL-C. By contrast, the heritability estimates after medication adjustments for HDL-C and TG stayed similar in both races. The heritability estimates for the adjusted lipid levels were similar to those when excluding medicated participants, suggesting that the medication adjustment may recover the phenotypic correlations within pedigrees previously obscured by medication.

**Table 4:** The effect of medication correction on heritability of lipids: the HyperGEN study

**Phenotype**	**Excluding medicated subjects**			**Including medicated subjects**					
		
	**N**	**Heritability**	**SE**	**N**	**Unadjusted with medications**		**Adjusted with medications**		**Significance**
							
					**Heritability**	**SE**	**Heritability**	**SE**	
**African**									
**Americans**									
**TC**	2088	0.57	0.07	2184	0.49	0.06	0.59	0.06	*
**HDL-C**	2080	0.30	0.05	2177	0.28	0.05	0.29	0.05	
**LDL-C**	2067	0.65	0.07	2162	0.55	0.06	0.69	0.07	*
**TG**	2088	0.50	0.05	2183	0.48	0.06	0.50	0.05	
**Whites**									
**TC**	2092	0.52	0.06	2391	0.36	0.04	0.51	0.05	*
**HDL-C**	2086	0.47	0.06	2385	0.50	0.05	0.48	0.05	
**LDL-C**	2034	0.53	0.06	2319	0.37	0.04	0.47	0.05	*
**TG**	2104	0.44	0.06	2390	0.32	0.04	0.34	0.04	

* The estimates of heritability are significantly different with and without medication adjustment.

BMI = Body Mass Index; TC = Total Cholesterol; HDL-C = High Density Lipoprotein Cholesterol; LDL-C = Low Density Lipoprotein Cholesterol; TG = Triglycerides.

A lod score of 1.75 (P < 0.0023) was set *a priori *as the threshold for promising linkages, which previously was shown to reduce the risk of type II errors to one false positive per genome scan (based on the assumption of discrete marker density with approximately 400 markers) [27]. Accordingly, all promising lod scores from the multipoint variance components linkage analysis for three samples, are presented in Table 5. Genome scan results of the four lipid traits for three groups of data are depicted in Figures A1-4 [see Additional File 1 Appendix A]. The strongest linkage signal (lod score = 3.08) was obtained for TG in African Americans excluding medicated subjects. This was localized at 131 cM from p-telomere (pter) on chromosome 11 (near marker AFM157XH6). The lod score dropped to 2.73 when medicated subjects were included without adjustment, and dropped to 2.71 when data were adjusted for the use of lipid-lowering medications. In contrast, the peak for TC on chromosome 6 at the region 53.81 cM p-ter (near marker GGAA15B08) increased in African Americans from 2.18 to 2.66 after the medication adjustment.

**Table 5:** Genome scan results (lod score ≥ 1.75) for lipids with and without medication correction by ethnicity: the HyperGEN study

				**Excluding medicated**		**Including medicated**		
					
**Phenotype**	**Chromosome**	**cM (from p-ter)**	**Marker**	**No. of sibs**	**Lod score**	**No. of sibs**	**Lod score**	
							
							**Medication unadjusted**	**Medication adjusted**
**African Americans**								
**TC**	6	53.81	GGAA15B08	1803	**2.03**	1889	**2.18**	**2.66**
	21	36.77	ATA27F01		**2.58**		**2.02**	**2.15**
	22	4.06	AFM217XF4		**1.95**		**1.82**	**2.04**
**HDL-C**	9	75.88	GATA89A11	1797	1.44	1884	**2.11**	**2.07**
**LDL-C**	2	132.58	GATA27A12	1786	1.46	1871	1.62	**1.79**
	6	73.13	GATA11E02		**2.22**		**2.36**	**1.81**
	21	57.77	GATA70B08		**1.73**		**1.93**	**1.77**
	22	4.06	AFM217XF4		1.50		**1.80**	**1.83**
**TG**	6	73.13	GATA11E02	1803	**1.89**	1888	**2.18**	**2.06**
	9	44.28	GATA87E02		**2.00**		1.05	0.93
	11	131.26	AFM157XH6		**3.08**		**2.73**	**2.71**
**Whites**								
**TC**	5	172.13	GATA7H10	1682	0.98	1924	0.73	**1.92**
	14	91.62	GATA30A03		0.58		**2.07**	0.44
**HDL-C**	1	274.53	GATA50F11	1678	**1.92**	1919	1.00	0.84
	8	0.73	AFM143XD8		1.24		**2.19**	1.67
	20	32.94	GATA81E09		1.72		**1.02**	**1.76**
**LDL-C**	9	88.92	GATA81C04	1682	**1.80**	1858	0.43	0.59

BMI = Body Mass Index; TC = Total Cholesterol; HDL-C = High Density Lipoprotein Cholesterol; LDL-C = Low Density Lipoprotein Cholesterol; TG = Triglycerides

## Discussion

The purpose of this study was to propose and explore a method to adjust blood lipid measurements for the effects of anti-hyperlipidemic medications. To our knowledge, our empirical approach to adjust medication effects on lipids has not been applied before in the genetic epidemiology studies, although examples of other complex traits exist. Several approaches have been attempted to adjust blood pressure for the use of antihypertensive medications. We reported a similar empirical method to adjust blood pressure based on a summary of over 160 clinical trials on effects of anti-hypertensive medication [28]. A nonparametric algorithm by ranking (the adjusted) blood pressure was implemented by Levy et al. in the Framingham Heart Study [29]. Hunt et al. compared three methods, including the use of a threshold method assigning a fixed blood pressure (140/90 mm Hg) for all medicated participants, and a random assignment of systolic blood pressure between 140–160, and a random assignment of diastolic blood pressure between 90–100 mm Hg [30]. The investigators concluded that excluding medicated measurements or the use of unadjusted blood pressure both lowered lod score results as compared to adjusting blood pressure measurements. Cui et al. examined the adjustment effects by adding an arbitrary 10 mm Hg and 5 mm Hg to systolic and diastolic blood pressures respectively, according to a publication on clinical trials [31,32]. The adjusted blood pressure resulted in an increase of both genetic and shared environmental variance components, while the unadjusted blood pressure for antihypertensive treatments obscured the relationship among phenotypes and genotypes.

The heritabilities of lipids, estimated in our study by excluding all the participants who took lipid-lowering medications, should be less biased heritabilities. However, by excluding subjects there is a smaller sample size and some valuable genetic information may be lost, especially alleles of the subjects affected more by dyslipidemia. Including medicated participants would introduce bias, because of a confounding of the medication effects with the lipid levels. That is, the measured lipid levels are artificial. This could cause a reduction in the genetic variance component as was seen for TC and LDL-C when we used the whole sample without performing medication adjustments. Heritabilities for TC and LDL-C were significantly improved after medication adjustments to the similar level as those obtained when excluding the medicated subjects. This indicates that the adjustment with our empirical method recovered relationships among phenotypes and genotypes obscured by the use of medications. Therefore, our method can provide the benefit of a greater power in genetic analysis by increasing the sample size as well as the information content.

The lod score for the TC peak in African Americans, centered at 53.81 cM on chromosome 6, increased from 2.03 to 2.66 when the medication adjustments were applied. This linkage signal is about 25 cM from a suggestive signal which was reported in a bivariate analysis of TG and LDL-C (LOD = 2.0) from 1,747 African American siblings in the Hypertension Genetic Epidemiology Network study [33]. Another study provided evidence of linkage (LOD = 2.14) at 102 cM on chromosome 6 for combined hyperlipidemia of TC and TG in 113 Caucasian families [5]. In contrast, the peak with a lod score of 3.08 dropped slightly to 2.71 when including those measures with medication adjustments for TG in African Americans on chromosome 11 at 131 cM from p-ter. This peak replicates a suggestive signal (LOD = 1.93) detected in French Canadians [6], and 30 cM from the region (LOD = 3.34) that have previously been identified in Turkish families [34]. We performed meta-analysis across African American and Caucasian families but there was no improvement in the LOD scores (results not shown).

Blood lipid levels are the result of a complex interaction among genetic and various environmental factors. The power of genome scans for detecting true signals is generally low with small-to-modest effect for complex traits like blood lipids. In addition, lipid-lowering treatment represents a pharmacologically induced alteration – typically of large magnitude – of the plasma lipid levels which further reduces the power to detect the true signals. One of the methods to overcome the confounding of medication effects with the measured lipid levels is to adjust the measured blood lipids by trying to recover the untreated levels as closely as possible. Although we noticed that the lod scores of blood lipids went both ways before and after medication adjustment in the present study, we expect lod scores for the medication adjusted analysis to be closest to their optimal (true) estimates. To evaluate our empirical approach of adjusting the treated lipid phenotypes, the heritability is a more appropriate parameter, since it reflects the effect size in linkage analysis. The higher heritabilities for TC and LDL-C when medication adjustments were made in the present study indicate an improvement in effect size of the linkage analysis with this empirical method. This is consistent with what is known about the HMG-CoA reductase inhibitors. First, as shown in Table 1 most of the medicated participants in the HyperGEN study took HMG-CoA reductase inhibitors and the effects is strongest on LDL-C and TC as shown in Table 3. Second, these inhibitors primarily target LDL-C (and by extension TC). For example, Table 2 shows larger mean and variance effects due to medication use for LDL-C and TC in the clinical trials literature.

Although our study has accounted for all participants and adjusted lipid measurements for aggregate medication effects, nevertheless there are limitations in the present study. The effects of anti-hyperlipidemics were estimated as group means rather than as individual responses to the medications, which are notoriously dose-dependent. Instead of producing precise untreated phenotype measurements, an adjustment with the average effects of medications may further serve as a shift by a constant and obscure the association between the phenotype and genotype. Also, we focused only on two groups of lipid-lowering medications, i.e., the most commonly used by subjects in the HyperGEN study. Under different circumstances an overall weighted average effect of other groups of anti-hyperlipidemic medications could be used. Another issue is that the present study did not ascertain the effect of the different patient groups which may influence antihyperlipidemic responses. Although dose-specific effects should be estimated when the information is available, the doses used in each of the source clinical trials were pooled to estimate the general efficacy for each group of medications because we do not have all the desired data in the HyperGEN study, such as dosage of anti-hyperlipidemics used by subjects. Similarly, we had to assume that the medicated subjects in the HyperGEN study had been prescribed, and were adhering, to the most commonly recommended doses. However, various strategies have been implemented to improve the compliance significantly during the clinical trials. The efficacy estimated from clinical trials is likely to overestimate the results observed in a general population of hyperlipidemic individuals under the treatment, such as in the HyperGEN study. Therefore, applying those estimates of drug effects would tend to over adjust the lipid values for studies using general populations.

## Conclusion

In conclusion, this study provides a summary of a large number of clinical trials on the average effects of anti-hyperlipidemic medications on the actual lipid phenotypes, and an empirical method to adjust measured lipids for such medication effects. An illustrative example of how to apply this methodology is presented for the HyperGEN data. The medication adjustments using this method resulted in an improvement in the heritability estimates for TC, LDL-C, and TG. Genome scans with the imputed lipid measurements revealed some promising regions, such as a QTL with a peak lod score 2.66 at 53.81 cM from p-ter on chromosome 6 for TC and a region with lod score of 2.71 centered at 131 cM on chromosome 11 for TG in African Americans. The method described and the medication-use adjustments derived from a large body of clinical trials can be employed in other epidemiologic and genetic studies to obtain more accurate estimates of the lipid measurements as well to preserve the power of the study.

## Authors' contributions

All authors contributed equally. All authors read and approved the final manuscript.

## Supplementary Material

Additional file 1 Appendix A and Appendix B. Appendix A includes Table A1 and A2 showing the detailed results from individual clinical trials for HMG-CoA Inhibitors and Fibric Acid Derivatives as mono-drug therapy, along with Figure A1-A4 for multipoint genome scan results of lipids in HyperGEN based on SEGPATH software. Appendix A may also be found on the following website: . Appendix B lists the citations for all clinical trials summarized in Table A1 and A2. Click here for file
