# Supplementary material for: An investigation of the effects of lipid-lowering medications: genome-wide linkage analysis of lipids in the HyperGEN study
Source: BMC Genet. 2007 Sep 10;8:60. doi: 10.1186/1471-2156-8-60 (PMC2045675; doi:10.1186/1471-2156-8-60)
Supplement: Additional file 1 — Appendix A and Appendix B. Appendix A includes Table A1 and A2 showing the detailed results from individual clinical trials for HMG-CoA Inhibitors and Fibric Acid Derivatives as mono-drug therapy, along with Figure A1-A4 for multipoint genome scan results of lipids in HyperGEN based on SEGPATH software. Appendix A may also be found on the following website: . Appendix B lists the citations for all clinical trials summarized in Table A1 and A2. [file 1471-2156-8-60-S1.pdf]

An investigation of the effects of lipid-lowering medications: genome-wide linkage analysis of lipids in the HyperGEN study

Jun Wu[1§], Michael A. Province[1], Hilary Coon[2], Steven C. Hunt[3], John H. Eckfeldt[4], Donna K. Arnett[5], Gerardo Heiss[6], Cora E. Lewis, R[7]. Curtis Ellison[8], D.C. Rao[9], Treva Rice[9], Aldi T. Kraja[1]

[1]Division of Statistical Genomics, Washington University School of Medicine, Campus Box 8506, 4444 Forest Park Boulevard, Saint Louis, MO63108, USA
[2]Department of Psychiatry, University of Utah, Salt Lake City, UT, USA
[3]Cardiovascular Genetics, University of Utah, Salt Lake City, UT, USA
[4]Department of Laboratory Medicine & Pathology, University of Minnesota, Minneapolis, MN, USA
[5]University of Alabama at Birmingham, Birmingham, AL, USA
[6]Department of Epidemiology, University of North Carolina, Chapel Hill, NC, USA
[7]Division of Preventive Medicine, University of Alabama at Birmingham, Birmingham, AL, USA
[8]Section of Preventive Medicine and Epidemiology, Boston University School of Medicine, Boston, MA, USA
[9]Division of Biostatistics, Washington University School of Medicine, Saint Louis, MO, USA

[§]Corresponding author

E-mail addresses:

JW: jwu@wustl.edu

MAP: mprovince@wustl.edu

HC: hilary.coon@m.cc.utah.edu

SCH: steve@ucvg.med.utah.edu

JHE: eckfe001@umn.edu

DKA: arnett@ms.soph.uab.edu

GH: gerardo_heiss@unc.edu

CEL: clewis@dopm.uab.edu

RCE: ellison@bu.edu

DCR: rao@wubios.wustl.edu

TR: Treva@wubios.wustl.edu

ATK: aldi@wustl.edu

Appendix A

Detailed results from individual clinical trials for HMG-CoA Inhibitors and Fibric Acid Derivatives as mono-drug therapy are summarized into 2 tables, numbered as Table A1 and A2 for mono-drug therapy. These tables, along with the overview of multipoint genome scan results based on SEGPATH software, for lipids in HyperGEN numbered as Figure A1-A4, may be found on the following website: http://www.biostat.wustl.edu/hypergen/results.html.

Figure legends:

Figure A1. Genome scan results for TC.

Figure A2. Genome scan results for HDL-C.

Figure A3. Genome scan results for LDL-C.

Figure A4. Genome scan results for TG.

Table A1. Summary of Clinical Trials for HMG-CoA Inhibitors

Author	Journal	Baseline (mg/dl)	Age (years)	Duration (weeks)	Ethnicity/ Country	No. of pts	Drug	Dosage (mg/day)	Lipid Level Change (mg/dl)			
									TC↓	LDL-C↓	HDL-C↑	TG ↓
Fong RL, et al.	AM J Med (1997)	TC 258<br>LDL-C 180<br>HDL-C 56<br>TG 119	53	10	Blacks	22	Lovastatin	20	42.3	38.7	1.0	18.8
Prisant LM, et al.	Am J Cardiol (1996)	TC 259<br>LDL-C 182<br>HDL-C 50<br>TG 120	55	48	Blacks	96	Lovastatin	20	36.9	34.7	1.5	16.73
Prisant LM, et al.	Am J Cardiol (1996)	TC 259<br>LDL-C 182<br>HDL-C 50<br>TG 120	55	48	Blacks	82	Lovastatin	40	49.1	45.6	1.5	22.7
Prisant LM, et al.	Am J Cardiol (1996)	TC 259<br>LDL-C 182<br>HDL-C 50<br>TG 120	55	48	Blacks	91	Lovastatin	20 bid	56.9	52.9	2.0	20.3
Prisant LM, et al.	Am J Cardiol (1996)	TC 259<br>LDL-C 182<br>HDL-C 50<br>TG 120	55	48	Blacks	99	Lovastatin	40 bid	72.4	67.5	1.5	25.1
Jacobson TA, et al.	Arch Intern Med (1995)	TC 281<br>LDL-C 210<br>HDL-C 44<br>TG 132	18~75	12	Blacks	182	Pravastatin	20	47.0	44.0	-2.0	14.0
Schweitzer M, et al.	Atherosclerosis (2002)	TC 240<br>LDL-C 155<br>HDL-C 46<br>TG 195	58	16	Caucasian 78%	70	Pravastatin	40	52.2	50.3	2.7	23.9
Downs JR, et al.	JAMA (1998)	TC 221<br>LDL-C 150<br>HDL-C 38<br>TG 158	45~73	52	White 89%<br>Black 3%<br>Hispanic 7%	2934	Lovastatin	20~40	44.0	41.0	1.0	20.0
The long-Term intervention with pravastatin in ischaemic disease	N Engl J Med (1998)	TC 218<br>LDL-C 150<br>HDL-C 36<br>TG 142	31~75	260	Australia<br>New Zealand	4512	Pravastatin	40	39.2	37.5	1.8	15.6
Stein E	Am J Cardiol (1998)	TC 290<br>LDL-C 208<br>HDL-C 48<br>TG 164	55	24	Whites 91%<br>Blacks 3%<br>Other 5%	206	Simvastatin	40	84.1	79.0	2.9	27.9
Stein E	Am J Cardiol (1998)	TC 289<br>LDL-C 205<br>HDL-C 48<br>TG 165	54	24	Whites 90%<br>Blacks 6%<br>Other 3%	311	Simvastatin	80	101.2	94.3	2.9	41.3

Table A1. Summary of Clinical Trials for HMG-CoA Inhibitors (Continued)

Author	Journal	Baseline (mg/dl)	Age (years)	Duration (weeks)	Ethnicity/ Country	No. of pts	Drug	Dosage (mg/day)	Lipid Level Change (mg/dl)			
									TC↓	LDL-C↓	HDL-C↑	TG ↓
Dart A, et al.	Am J Cardiol (1997)	TC 290<br>LDL-C 212<br>HDL-C 42<br>TG 183	18~80	52	Australia	132	Atorvastatin	20	87.3	81.3	2.9	38.9
Dart A, et al.	Am J Cardiol (1997)	TC 290<br>LDL-C 212<br>HDL-C 42<br>TG 183	18~80	52	Australia	45	Simvastatin	20	71.0	68.6	2.8	21.6
Sacks FM, et al.	N Engl J Med (1996)	TC 209<br>LDL-C 139<br>HDL-C 39<br>TG 156	21~75	260	White 93%<br>Other 7%	2081	Pravastatin	40	41.8	38.9	2.0	22.1
Shepherd J, et al.	N Engl J Med (1995)	TC 272<br>LDL-C 192<br>HDL-C 44<br>TG 162	45~64<br>Men	260	Scotland	3302	Pravastatin	40	54.4	49.9	2.2	19.4
Scandinavian Simvastatin Survival Study Group	Lancet (1994)	TC 260<br>LDL-C 188<br>HDL-C 46<br>TG 132	35~70	280	Scandinavian	2221	Simvastatin	20~40	67.7	67.7	3.1	22.1
Valles F, et al. (Group I)	Atherosclerosis (1991)	TC 274<br>LDL-C 202<br>HDL-C 46<br>TG 132	54	12	Spain	44	Lovastatin	20	56.0	57.0	4.0	22.0
Crepaldi G, et al.	Arch Intern Med (1991)	TC 354<br>LDL-C 280<br>HDL-C 48<br>TG 132	18~70	24	Italian	193	Pravastatin	40	79.0	83.0	2.0	11.0
Tikkanen MJ, et al. (Stratum I)	Am J Med (1989)	TC 296<br>LDL-C 217<br>HDL-C 46<br>TG 165	52	12	White 64%<br>Metizo 4%<br>Other 1%	68	Simvastatin	5~10	56.8	48.6	2.9	26.7
Tikkanen MJ, et al. (Stratum II)	Am J Med (1989)	TC 347<br>LDL-C 265<br>HDL-C 49<br>TG 151	51	12	White 72%<br>Metizo 4%<br>Other 2%	78	Simvastatin	10~20	93.3	89.6	4.5	10.7
Valles F, et al. (Group II)	Atherosclerosis (1991)	TC 364<br>LDL-C 284<br>HDL-C 51<br>TG 147	53	12	Spain	42	Lovastatin	40	96.0	95.0	3.0	17.0

Table A2. Summary of Clinical Trials for Fibric Acid Derivatives

Author	Journal	Baseline (mg/dl)	Age (years)	Duration (weeks)	Ethnicity/ Country	No. of pts	Drug	Dosage (mg/day)	Lipid Level Change (mg/dl)			
									TC↓	LDL-C↓	HDL-C↑	TG ↓
Schweitzer M, et al.	Atherosclerosis (2002)	TC 240<br>LDL-C 155<br>HDL-C 46<br>TG 195	57	16	Caucasian 82%	66	Gemfibrozil	1200	16.2	8.5	2.7	68.2
Rubins HB, et al.	N Engl J Med (1999)	TC 175<br>LDL-C 111<br>HDL-C 32<br>TG 161	64	265	White 90%<br>Black 8%<br>Other 2%	1264	Gemfibrozil	1200	7.0	2.0	2.0	52.0
Knipscheer HC, et al.	Atherosclerosis (1996)	TC 346<br>LDL-C 262<br>HDL-C 47<br>TG 213	21~75	12	Netherlands	45	Gemfibrozil	1200	50.3	37.9	10.4	97.4
Schaefer EJ, et al. (Trial 1)	Atherosclerosis (1996)	TC 290<br>LDL-C 209<br>HDL-C 35<br>TG	52	12	White 90%<br>Black 8%<br>Other 2%	111	Gemfivrozil MR	1200	24.0	6.2	3.1	74.4
Schaefer EJ, et al. (Trial 2)	Atherosclerosis (1996)	TC 286<br>LDL-C 205<br>HDL-C 35<br>TG	53	26	White 91%<br>Black 6%<br>Other 3%	330	Gemfivrozil IR	1200	26.7	9.7	3.1	72.6
Schaefer EJ, et al. (Trial 2)	Atherosclerosis (1996)	TC 286<br>LDL-C 205<br>HDL-C 35<br>TG 177	53	26	White 91%<br>Black 6%<br>Other 3%	325	Gemfivrozil MR	1200	25.5	9.7	3.1	68.2
Crepaldi G, et al.	Arch Intern Med (1991)	TC 350<br>LDL-C 272<br>HDL-C 49<br>TG 140	18~70	24	Italian	192	Gemfibrozil	1200	49.0	43.0	5.0	57.0
Valles F, et al. (Group I)	Atherosclerosis (1991)	TC 270<br>LDL-C 196<br>HDL-C 48<br>TG 130	53	12	Spain	43	Gemfibrozil	1200	20.0	19.0	7.0	38.0
Valles F, et al. (Group II)	Atherosclerosis (1991)	TC 345<br>LDL-C 266<br>HDL-C 46<br>TG 169	51	12	Spain	53	Gemfibrozil	1200	46.0	39.0	4.0	56.0
Tikkanen MJ, et al. (Stratum I)	Am J Med (1989)	TC 318<br>LDL-C 240<br>HDL-C 46<br>TG 165	52	12	White 64%<br>Metizo 4%<br>Other 0%	69	Gemfibrozil	1200	44.2	41.5	4.6	49.7
Tikkanen MJ, et al. (Stratum II)	Am J Med (1989)	TC 350<br>LDL-C 272<br>HDL-C 46<br>TG 159	50	12	White 71%<br>Metizo 4%<br>Other 0%	75	Gemfibrozil	1200	52.9	45.4	7.5	51.5

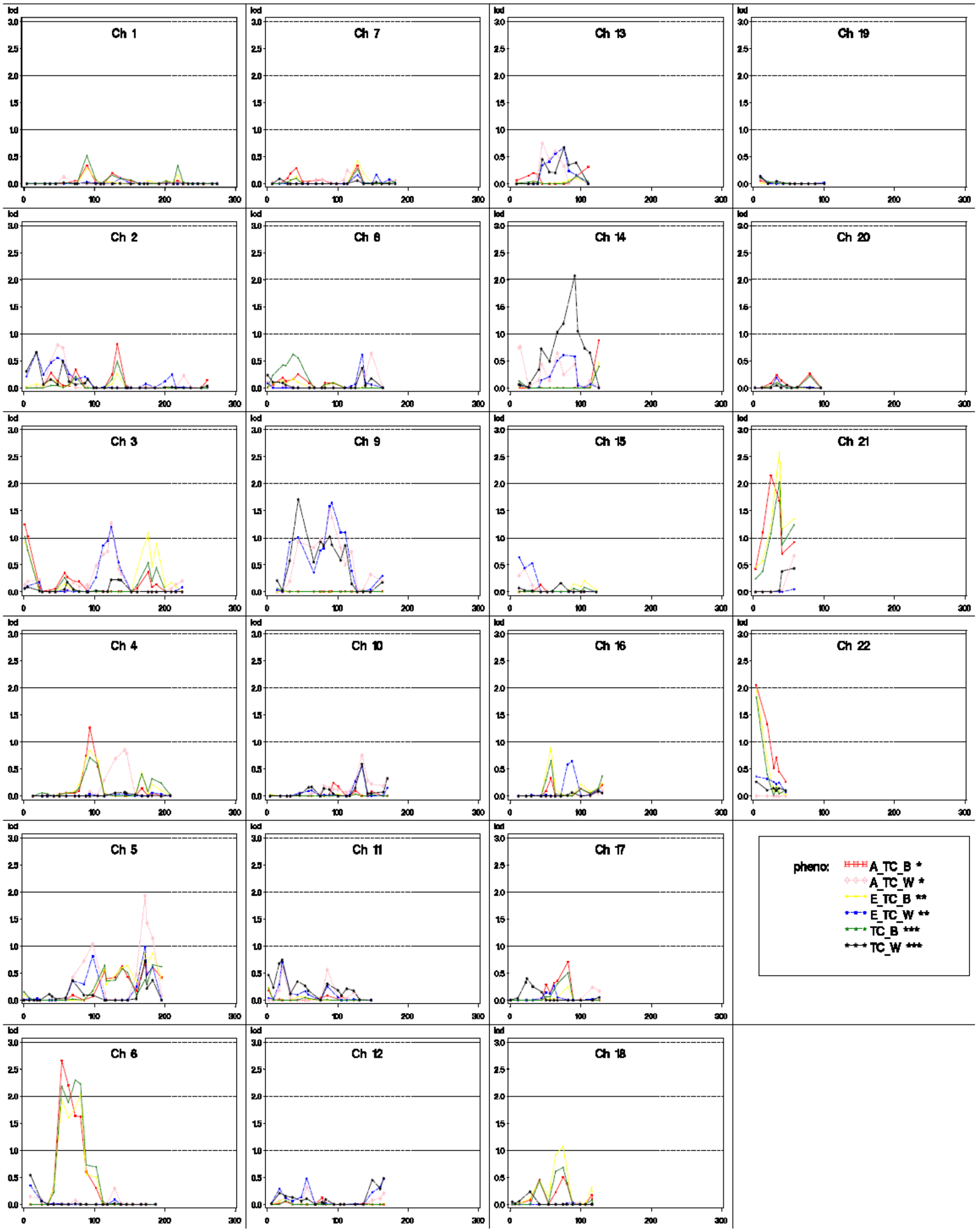

Fig A1. Genome scan results for TC.

* Phenotypes for all participants without any adjustment for medication effects
** Phenotypes when excluding medicated participants
*** Phenotypes for all participants with adjustment for medication effects

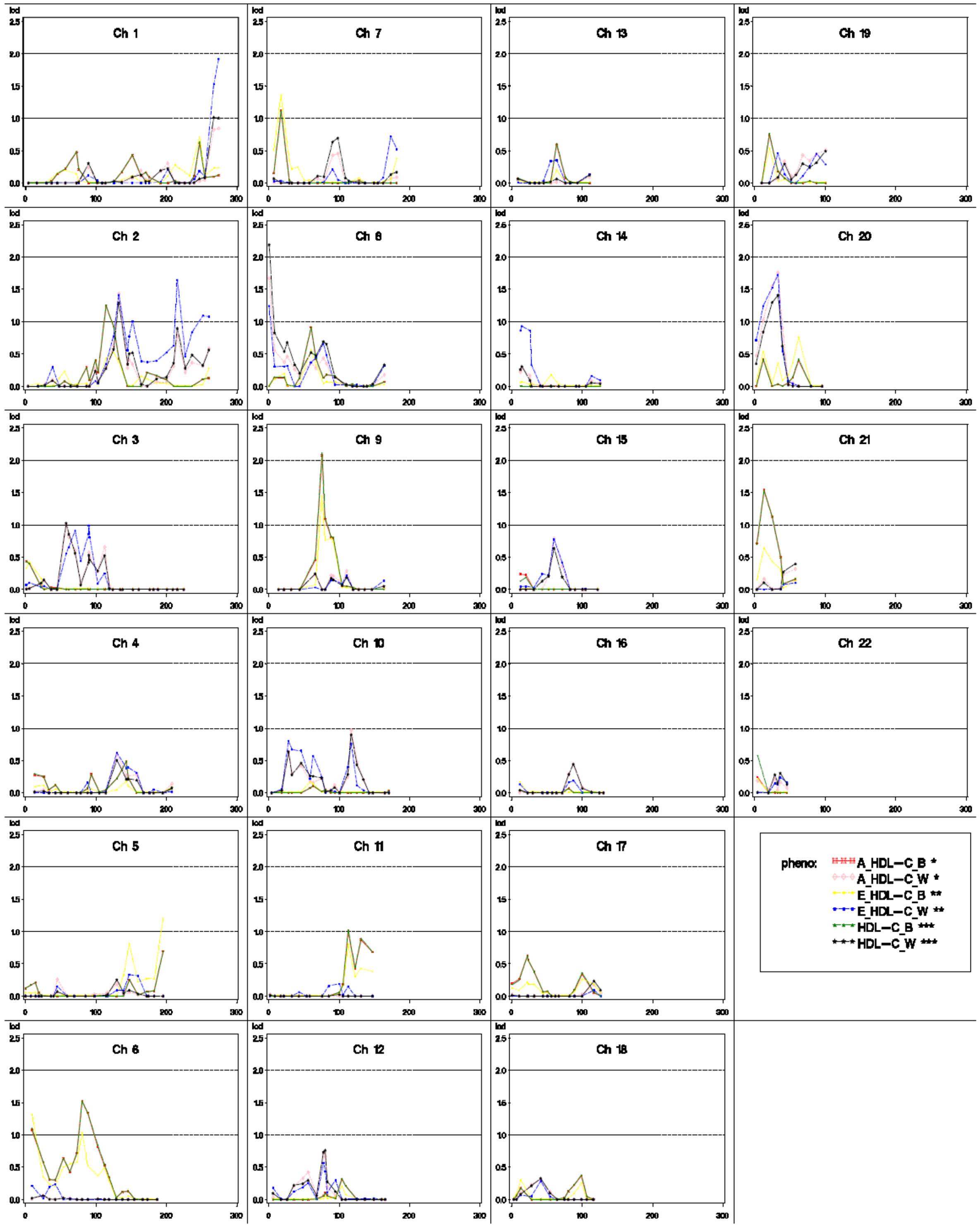

Fig A2. Genome scan results for HDL-C.

* Phenotypes for all participants with adjustment for medication effects
** Phenotypes when excluding medicated participants
*** Phenotypes for all participants without adjustment for medication effects

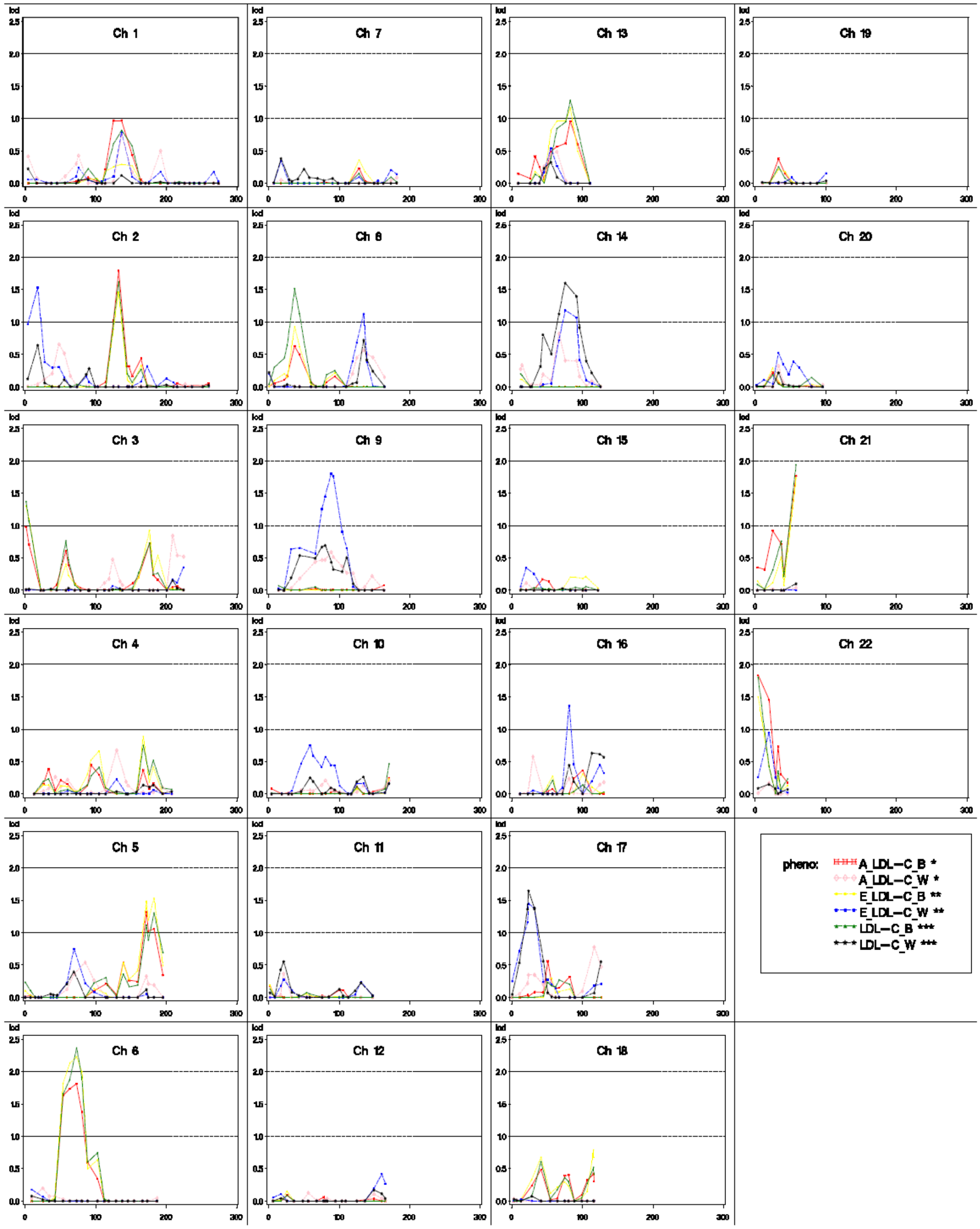

Fig A3. Genome scan results for LDL-C.

* Phenotypes for all participants with adjustment for medication effects

** Phenotypes when excluding medicated participants

*** Phenotypes for all participants without adjustment for medication effects

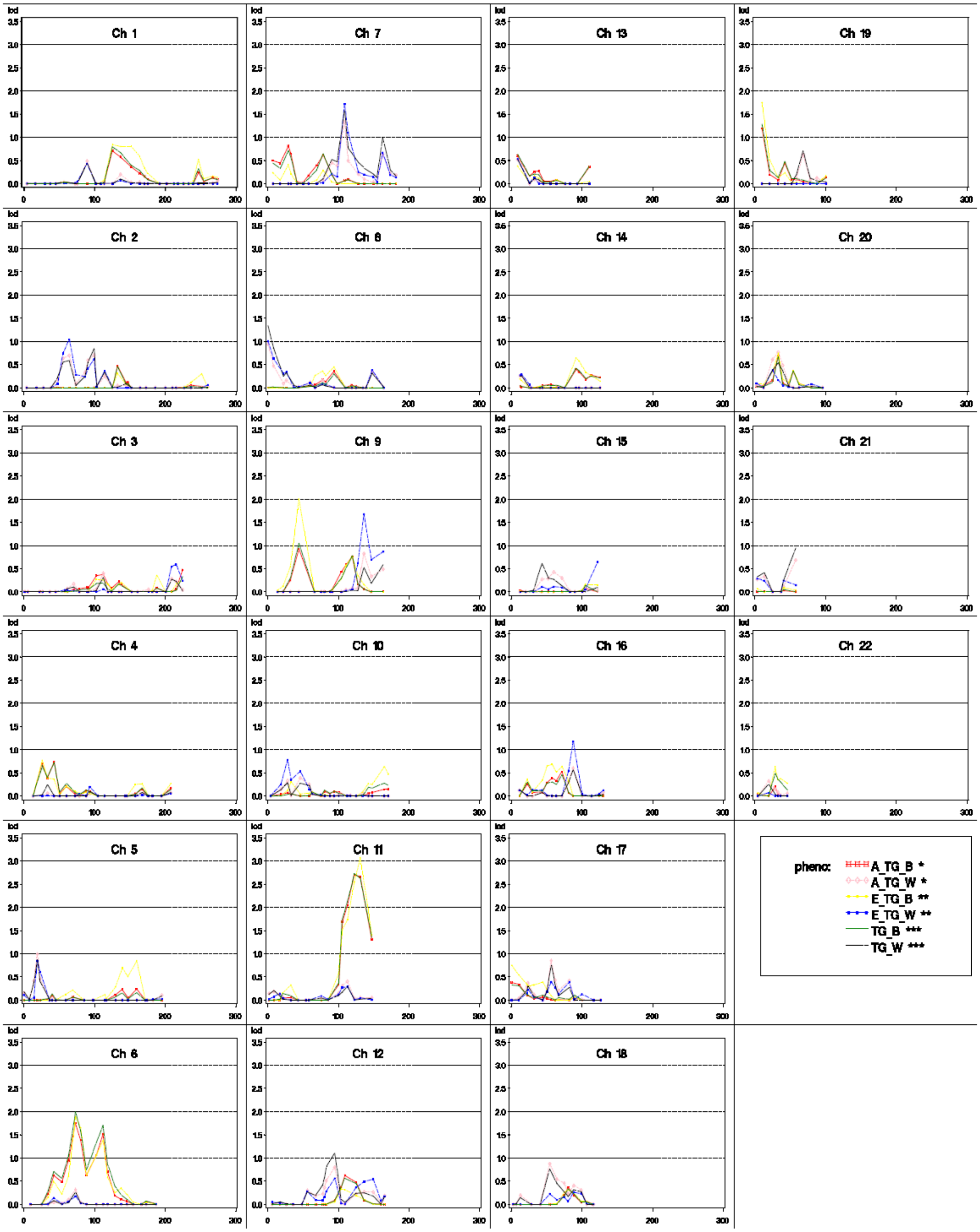

Fig A4. Genome scan results for TG.

*   Phenotypes for all participants with adjustment for medication effects
**   Phenotypes when excluding medicated participants
*** Phenotypes for all participants without adjustment for medication effects

Appendix B

Citations for all clinical trials summarized in Table A1 and A2.

Aguilar-Salinas CA, Fanghanel-Salmon G, Meza E, Montes J, Gulias-Herrero A, Sanchez L, Monterrubio-Flores EA, Gonzalez-Valdez H, Gomez Perez FJ.Ciprofibrate versus gemfibrozil in the treatment of mixed hyperlipidemias: an open-label, multicenter study. *Metabolism.* 2001;50:729-733.

Chong PH, Tzallas-Pontikes PJ, Seeger JD, Stamos TD. The low-density lipoprotein cholesterol-lowering effect of pravastatin and factors associated with achieving targeted low-density lipoprotein levels in an African-American population. *Pharmacotherapy.* 2000;20:1454-1463.

Crepaldi G, Baggio G, Arca M, Avellone G, Avogaro P, Bittolo Bon G, Bompiani GD, Capurso A, Cattin L, D'Alo G, et al. Pravastatin vs gemfibrozil in the treatment of primary hypercholesterolemia. The Italian Multicenter Pravastatin Study I. *Arch Intern Med.* 1991;151:146-152.

Dart A, Jerums G, Nicholson G, d'Emden M, Hamilton-Craig I, Tallis G, Best J, West M, Sullivan D, Bracs P, Black D. A multicenter, double-blind, one-year study comparing safety and efficacy of atorvastatin versus simvastatin in patients with hypercholesterolemia. *Am J Cardiol.* 1997;80:39-44.

Downs JR, Clearfield M, Weis S, Whitney E, Shapiro DR, Beere PA, Langendorfer A, Stein EA, Kruyer W, Gotto AM Jr. Primary prevention of acute coronary events with lovastatin in men and women with average cholesterol levels: results of AFCAPS/TexCAPS. Air Force/Texas Coronary Atherosclerosis Prevention Study. *JAMA.* 1998;279:1615-1622.

Valles F, Anguita M, Anglada J, Aguirre C, Fabiani F, Plaza L, Soriguer F, Azanza JR, Barcina C. A multicenter double-blind study comparing lovastatin and gemfibrozil in the treatment of primary hypercholesterolemia. *Atherosclerosis.* 1991;91 Suppl:S3-S9.

Fong RL, Ward HJ. The efficacy of lovastatin in lowering cholesterol in African Americans with primary hypercholesterolemia. *Am J Med.* 1997;102:387-391.

Jacobson TA, Chin MM, Curry CL, Miller V, Papademetriou V, Schlant RC, LaRosa JC. Efficacy and safety of pravastatin in African Americans with primary hypercholesterolemia. *Arch Intern Med.* 1995;155:1900-1906.

Knipscheer HC, de Valois JC, van den Ende B, Wouter ten Cate J, Kastelein JJ. Ciprofibrate versus gemfibrozil in the treatment of primary hyperlipidaemia. *Atherosclerosis.* 1996;124 Suppl:S75-S81.

The Long-Term Intervention with Pravastatin in Ischaemic Disease (LIPID) Study Group.Prevention of cardiovascular events and death with pravastatin in patients with coronary heart disease and a broad range of initial cholesterol levels. *N Engl J Med.* 1998;339:1349-1357.

Prisant LM, Downton M, Watkins LO, Schnaper H, Bradford RH, Chremos AN, Langendorfer A.Efficacy and tolerability of lovastatin in 459 African-Americans with hypercholesterolemia. *Am J Cardiol.* 1996;78:420-424.

Rubins HB, Robins SJ, Collins D, Fye CL, Anderson JW, Elam MB, Faas FH, Linares E, Schaefer EJ, Schectman G, Wilt TJ, Wittes J. Gemfibrozil for the secondary prevention of coronary heart disease in men with low levels of high-density lipoprotein cholesterol. Veterans Affairs High-Density Lipoprotein Cholesterol Intervention Trial Study Group. *N Engl J Med.* 1999;341:410-418.

Sacks FM, Pfeffer MA, Moye LA, Rouleau JL, Rutherford JD, Cole TG, Brown L, Warnica JW, Arnold JM, Wun CC, Davis BR, Braunwald E.The effect of pravastatin on coronary events after myocardial infarction in patients with average cholesterol levels. Cholesterol and Recurrent Events Trial investigators. *N Engl J Med.* 1996;335:1001-1009.

Schaefer EJ, Lamon-Fava S, Cole T, Sprecher DL, Cilla DD Jr, Balagtas CC, Rowan JP, Black DM. Effects of regular and extended-release gemfibrozil on plasma lipoproteins and apolipoproteins in hypercholesterolemic patients with decreased HDL cholesterol levels. *Atherosclerosis.* 1996;127:113-122.

Schweitzer M, Tessier D, Vlahos WD, Leiter L, Collet JP, McQueen MJ, Harvey L, Alaupovic P. A comparison of pravastatin and gemfibrozil in the treatment of dyslipoproteinemia in patients with non-insulin-dependent diabetes mellitus. *Atherosclerosis.* 2002;162:201-210.

Shepherd J, Cobbe SM, Ford I, Isles CG, Lorimer AR, MacFarlane PW, McKillop JH, Packard CJ. Prevention of coronary heart disease with pravastatin in men with hypercholesterolemia. West of Scotland Coronary Prevention Study Group. *N Engl J Med.* 1995;333:1301-1307.

Stein EA, Davidson MH, Dobs AS, Schrott H, Dujovne CA, Bays H, Weiss SR, Melino MR, Stepanavage ME, Mitchel YB. Efficacy and safety of simvastatin 80 mg/day in hypercholesterolemic patients. The Expanded Dose Simvastatin U.S. Study Group. *Am J Cardiol.* 1998;82:311-316.

Tikkanen MJ, Bocanegra TS, Walker JF, Cook T. Comparison of low-dose simvastatin and gemfibrozil in the treatment of elevated plasma cholesterol. A multicenter study. The Simvastatin Study Group. *Am J Med.* 1989;87:47S-53S

Wagner AM, Jorba O, Bonet R, Ordonez-Llanos J, Perez A. Efficacy of atorvastatin and gemfibrozil, alone and in low dose combination, in the treatment of diabetic dyslipidemia. *J Clin Endocrinol Metab.* 2003;88:3212-3217.
